# Hidden Gut Dysbiosis in Apparently Healthy Adults

**DOI:** 10.3390/life16091425

**Published:** 2026-08-27

**Authors:** Valentina Avram, Klavio Pine, Nicoleta Negrut, Anca Ferician, Paula Marian

**Affiliations:** 1 Doctoral School of Biomedical Sciences, Faculty of Medicine and Pharmacy, University of Oradea, 410087 Oradea, Romania; avram.valentinaelena@student.uoradea.ro; 2 Department of Infectious Diseases, Faculty of Medicine, “Vasile Goldiș” Western University of Arad, 310025 Arad, Romania; 3 Department of Psycho-Neuroscience and Recovery, Faculty of Medicine and Pharmacy, University of Oradea, 410073 Oradea, Romania; 4 Department of Medical Disciplines, Faculty of Medicine and Pharmacy, University of Oradea, 410073 Oradea, Romania; anca.ferician@uoradea.ro (A.F.); paulamarian@uoradea.ro (P.M.)

**Keywords:** intestinal dysbiosis, gut microbiota, asymptomatic adults, microbiota screening, mucosal immunity, fecal biomarkers

## Abstract

(1) Background: Intestinal dysbiosis has been implicated in numerous gastrointestinal, metabolic, and immune-mediated disorders; however, data regarding its prevalence in asymptomatic adults remain limited. This study evaluated the prevalence and integrated profile of microbiological, functional, and immunological alterations associated with dysbiosis in apparently healthy adults undergoing intestinal microbiota screening. (2) Methods: A laboratory-based cross-sectional study included 98 asymptomatic adults. In the present study, intestinal dysbiosis was defined as a multidimensional laboratory phenotype reflecting disruption of intestinal microbial, fungal, metabolic, luminal, or mucosal immune homeostasis. Bacterial and fungal markers, short-chain fatty acids (SCFAs), fecal pH, and mucosal immune biomarkers were assessed and grouped into predefined biological domains. Dysbiosis complexity was evaluated according to the number of affected domains. (3) Results: At least one laboratory abnormality was identified in 97 participants (99.0%), while 54 (55.1%) exhibited alterations in three or more biological domains. Core bacterial imbalance was detected in 87 (88.8%) participants, inflammatory and mucosal immune abnormalities in 85 (86.7%), opportunistic bacterial overgrowth in 61 (62.2%), depletion of protective bacteria in 58 (59.2%), and fungal overgrowth in 49 (50.0%). Reduced SCFA markers were observed in 27 (27.6%) participants and altered fecal pH in 33 (33.7%). Correlation analysis revealed clustering of SCFA markers and biologically plausible associations between microbial and mucosal immune biomarkers. (4) Conclusions: Laboratory abnormalities related to intestinal dysbiosis were highly prevalent and predominantly occurred as multidomain profiles, supporting dysbiosis as a complex biological phenotype requiring integrated microbiological, metabolic, and immunological assessment.

## 1. Introduction

The human gut microbiota is a complex and dynamic ecosystem that interacts closely with the host and contributes to intestinal and systemic homeostasis through multiple mechanisms, including nutrient metabolism, epithelial barrier maintenance, colonization resistance, mucosal immune regulation, and modulation of inflammatory pathways [1,2,3,4]. Alterations in gut microbiota composition and function have been associated with gastrointestinal, metabolic, and immune-mediated disorders. Intestinal dysbiosis is generally understood as a disruption of gut microbial ecosystem homeostasis, involving compositional, functional, metabolic, or host–microbiota interaction abnormalities rather than a single isolated microbial alteration [2,3,4].

Protective bacteria, particularly *Lactobacillus* spp. and *Bifidobacterium* spp., play an important role in maintaining intestinal homeostasis through colonization resistance, epithelial barrier support, and immune modulation [5,6,7]. Their depletion, often accompanied by expansion of facultative organisms such as Escherichia coli and other Enterobacteriaceae, may indicate intestinal dysbiosis [5,6,7].

Microbial metabolites, particularly SCFAs, contribute to intestinal barrier integrity, energy metabolism, and immune regulation [5]. Reduced SCFA levels and alterations in fecal pH may therefore reflect functional disturbances of the gut ecosystem.

The intestinal mycobiota also contributes to gut ecosystem stability. Although *Candida* spp. may occur as commensals, excessive fungal growth may be associated with impaired microbial balance and altered host–microbiota interactions [8,9,10,11].

Host-derived markers such as calprotectin, β-defensin, eosinophil protein X (EPX), and secretory IgA (sIgA) may provide complementary information regarding mucosal immune responses associated with dysbiosis-related abnormalities.

Most research on intestinal dysbiosis has focused on symptomatic individuals or patients with established gastrointestinal disorders, particularly inflammatory bowel disease [12,13,14,15,16]. Data regarding dysbiosis-related abnormalities in asymptomatic adults remain limited. Given the substantial interindividual variability of the gut microbiota, influenced by factors such as age, diet, medication use, metabolic status, and lifestyle, the assessment of dysbiosis in apparently healthy individuals should rely on the overall pattern of microbiological alterations rather than on isolated findings [17,18,19].

Because no universally accepted laboratory definition of dysbiosis exists, particularly for culture-based testing, a multidimensional assessment is often more informative than isolated marker evaluation [18,20,21,22]. Therefore, this study investigated the prevalence, severity, and co-occurrence of dysbiosis-related abnormalities in asymptomatic adults, focusing on microbiological, metabolic, and mucosal immune markers.

## 2. Materials and Methods

### 2.1. Study Design and Population

This laboratory-based cross-sectional study evaluated microbiota-related laboratory abnormalities in asymptomatic adults undergoing stool microbiota screening between September 2024 and March 2025. Participants were recruited using a convenience sampling approach. Adults who voluntarily expressed interest in stool microbiota screening were identified through referrals from primary care physicians, collaborating clinical laboratories, and the investigators’ professional network. Individuals meeting the predefined eligibility criteria were invited to participate after receiving information about the study and providing written informed consent. Eligible participants were adults with complete microbiological, functional, and mucosal immune marker data who met the predefined inclusion and exclusion criteria.

Participants were considered asymptomatic if they reported no acute gastrointestinal symptoms at the time of stool sample collection. Accordingly, the study population was considered a screening cohort rather than a population with gastrointestinal disease.

The study was approved by the Ethics Committee of the Faculty of Medicine, Arad, Romania (Approval No. 266/28 August 2024). All participants provided written informed consent before enrollment. The study was conducted in accordance with the ethical principles of the Declaration of Helsinki.

### 2.2. Inclusion and Exclusion Criteria

Eligible participants met all of the following criteria:

- Age ≥ 18 years;
- Complete stool microbiota testing results;
- Complete functional and mucosal immune fecal marker results;
- Absence of self-reported acute gastrointestinal symptoms at the time of stool sample collection.

Exclusion criteria were as follows:

- Antibiotic, antifungal, or antiparasitic treatment within the previous 2–4 weeks;
- Probiotic use within 14 days before stool sample collection;
- Proton pump inhibitor or bismuth-containing compound use within 14 days before sampling;
- Use of nonsteroidal anti-inflammatory drugs, laxatives, enemas, or digestive supplements within 48 h before sampling;
- Colonoscopy or enema within the previous 4 weeks;
- Active gastrointestinal bleeding, including hemorrhoidal bleeding;
- Stool sample collection during menstruation;
- Incomplete or invalid stool sample submission.

These criteria were applied to minimize potential confounding related to medication exposure, gastrointestinal procedures, acute gastrointestinal events, and pre-analytical variability.

### 2.3. Patient Preparation and Sample Collection

Participants received standardized pre-analytical instructions before stool sample collection. Sample collection was performed in accordance with the laboratory protocol, and eligibility was verified against the predefined inclusion and exclusion criteria.

Each participant provided a single stool specimen, from which material was collected into three sterile stool collection containers without transport medium, each filled to approximately three-quarters of its volume, in accordance with the laboratory protocol. Participants were instructed to avoid contamination with urine, water, menstrual blood, or disinfectants. According to the laboratory pre-analytical requirements for intestinal microbiota analysis, stool specimens were considered stable for up to 2 days at ambient temperature.

Samples with inadequate collection or handling, or incomplete laboratory results, were excluded. Sample collection was performed individually during the recruitment period, according to each participant’s enrollment and scheduled laboratory submission.

### 2.4. Laboratory Analysis and Interpretation of Biomarkers

Stool samples were analyzed using standardized laboratory procedures to assess microbiological, fungal, metabolic, and mucosal immune parameters relevant to intestinal dysbiosis. Quantitative bacterial assessment, including *Enterococcus* spp., *Escherichia coli*, *Bacteroides* spp., *Enterobacteriaceae*, *Lactobacillus* spp., and *Bifidobacterium* spp., was performed using quantitative stool culture methods. Results were expressed as colony-forming units per gram of feces (CFU/g) and interpreted according to laboratory-specific reference intervals.

Opportunistic bacterial markers, including *Citrobacter* spp., *Enterobacter* spp., *Klebsiella* spp., *Proteus* spp., *Serratia* spp., *Pseudomonas* spp., alpha-haemolytic streptococci, beta-haemolytic streptococci, and *Enterobacteriaceae*, were also assessed using quantitative stool culture methods and classified according to laboratory-specific reference thresholds.

Fungal markers, including *Candida* spp. and *Candida albicans*, were assessed using quantitative stool culture methods. Results were expressed as CFU/g and interpreted according to laboratory-defined thresholds for fungal overgrowth.

Functional metabolic markers included fecal SCFAs, namely acetate, propionate, and butyrate. SCFA concentrations were determined by gas chromatography–tandem mass spectrometry, expressed as μmol/g of feces, and interpreted according to laboratory-specific reference thresholds. Fecal pH was measured as part of the laboratory microbiota assessment and interpreted according to the laboratory-specific reference interval of 5.5–6.5.

Mucosal immune and inflammatory biomarkers included fecal beta-defensin, calprotectin, EPX, and secretory IgA. All four biomarkers were measured using enzyme-linked immunosorbent assay. Results were expressed as ng/g or μg/g of feces, depending on the biomarker, and interpreted according to the corresponding laboratory-specific reference intervals.

The selected bacterial and fungal markers were chosen to represent complementary components of the intestinal ecosystem relevant to dysbiosis assessment. *Lactobacillus* spp. and *Bifidobacterium* spp. were included as protective commensal markers because of their roles in colonization resistance, intestinal barrier function, and mucosal immune regulation. *Bacteroides* spp. were included as representative core gut bacteria involved in intestinal microbial and metabolic homeostasis. *Enterococcus* spp., *Escherichia coli*, and *Enterobacteriaceae* were included as markers of intestinal microbial balance, while opportunistic genera, including *Citrobacter*, *Enterobacter*, *Klebsiella*, *Proteus*, *Serratia*, and *Pseudomonas*, were assessed to identify potential opportunistic overgrowth. *Candida* spp. and *Candida albicans* were included as fungal markers of altered bacterial–fungal ecological balance. Together, these markers were used to characterize protective bacterial depletion, core bacterial imbalance, opportunistic bacterial expansion, and fungal overgrowth as complementary laboratory domains of intestinal dysbiosis.

All laboratory results were interpreted according to the reference intervals and decision thresholds provided by the diagnostic laboratory. Parameter classification in Table 1 was based on whether the measured value fell below, within, or above the corresponding laboratory reference interval. Values within the reference interval were classified as normal. Values below the lower reference limit were classified as decreased or low, whereas values above the upper reference limit were classified as increased or high. For parameters defined by a single decision threshold, values exceeding the threshold were classified as abnormal or increased, according to the direction of clinical interpretation. These classifications were used for descriptive and domain-based analyses and were not intended to establish a clinical diagnosis by themselves.

### 2.5. Definition of Dysbiosis and Analytical Framework

For analytical purposes, intestinal dysbiosis was operationally defined as a multidimensional laboratory phenotype reflecting disruption of intestinal microbial, fungal, metabolic, luminal, or mucosal immune homeostasis. This phenotype was assessed using predefined dysbiosis domains, and individual marker abnormalities were interpreted within the broader pattern of domain-level involvement rather than as isolated disease-defining findings.

Marker-level abnormalities were grouped into predefined dysbiosis domains. A domain was considered present when at least one marker belonging to that domain was outside the corresponding reference interval. Core bacterial imbalance included abnormalities involving *Enterococcus* spp., *Escherichia coli*, *Bacteroides* spp., or *Enterobacteriaceae*. Protective bacterial depletion was defined as a decrease in *Lactobacillus* spp. and/or *Bifidobacterium* spp. Opportunistic overgrowth was defined as increased levels of *Citrobacter* spp., *Enterobacter* spp., *Klebsiella* spp., *Proteus* spp., *Serratia* spp., *Pseudomonas* spp., alpha-haemolytic streptococci, beta-haemolytic streptococci, and/or *Enterobacteriaceae*. Fungal overgrowth was defined as increased *Candida* spp. and/or *Candida albicans*. Functional abnormalities included decreased acetate, propionate, and/or butyrate levels and/or abnormal fecal pH. In contrast, inflammatory or immune abnormalities were assessed using beta-defensin, calprotectin, eosinophil protein X, and secretory IgA.

For each participant, the total number of dysbiosis domains present was calculated. Dysbiosis complexity was classified as mild (one altered domain), moderate (two altered domains), or complex (three or more altered domains).

The analytical approach included descriptive evaluation of marker-level abnormalities, dysbiosis domains, and dysbiosis complexity. Correlation analyses were performed to explore associations among microbiological, fungal, functional, and mucosal immune markers. Given the cross-sectional design, all findings were interpreted as associative rather than causal.

### 2.6. Statistical Analysis

Statistical analysis was performed using IBM SPSS Statistics version 30.0 (IBM Corp., Armonk, NY, USA). The normality of continuous variables was assessed using the Shapiro–Wilk test. Normally distributed variables were summarized as mean ± standard deviation (SD), whereas non-normally distributed variables were presented as median with interquartile range (IQR) and range. Quantitative analyses of biomarker concentrations were performed only for participants with values outside the corresponding laboratory reference intervals. Categorical variables were expressed as frequencies and percentages. Comparisons of dysbiosis domain prevalence according to sex and place of residence were performed using the Chi-square test or Fisher’s exact test, as appropriate. Spearman’s rank correlation analysis was used to assess associations between microbiota-related, functional, and mucosal immune markers. All tests were two-sided, and *p* < 0.05 was considered statistically significant.

## 3. Results

### 3.1. Study Population

**Figure 1:**
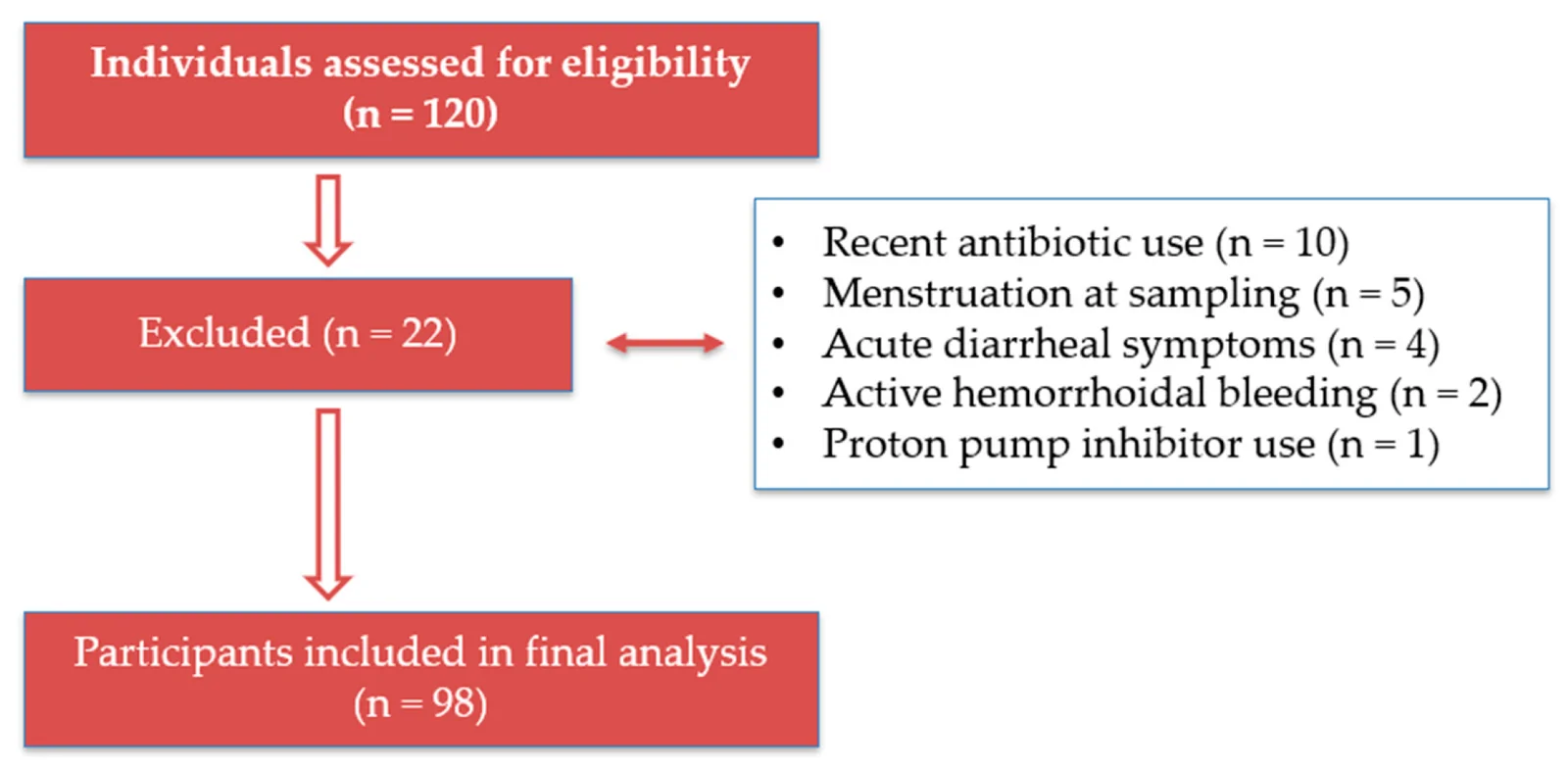
Flowchart of participant selection and inclusion.

A total of 120 individuals were assessed for eligibility. Following the application of the predefined inclusion and exclusion criteria, 98 asymptomatic adults were included in the final analysis (Figure 1). The study population had a mean age of 45.8 ± 12.7 years. Women represented 53 (54.08%) of participants, while 53 (54.08%) were from urban areas. Neither the sex distribution (χ^2^(1) = 0.65, *p* = 0.419) nor the urban–rural distribution (χ^2^(1) = 0.65, *p* = 0.419) differed significantly from an equal distribution.

Additional clinical, anthropometric, and lifestyle variables were not systematically collected and were therefore not included in the present analysis.

### 3.2. Prevalence of Microbiota Alterations

#### 3.2.1. Marker-Level Prevalence of Microbiota Alterations

Marker-level abnormalities were identified across bacterial, fungal, functional, and mucosal immune domains (Table 2). Among bacterial markers, the most frequently altered were *Enterococcus* spp., *Escherichia coli*, and *Lactobacillus* spp. Fungal overgrowth markers were also commonly detected.

Functional abnormalities included altered fecal pH and reduced SCFA markers, whereas mucosal immune abnormalities were primarily reflected in altered secretory IgA and beta-defensin levels. In contrast, EPX remained within the reference range in all participants.

#### 3.2.2. Quantitative Characteristics of Abnormal Biomarker Values

The distributions of abnormal biomarker concentrations were assessed using the Shapiro–Wilk test and were non-normal for all evaluated biomarkers. Accordingly, quantitative data are presented as median (IQR) and range. Median concentrations were 83 μg/g (IQR: 60–118 μg/g; range: 53–193 μg/g) for fecal calprotectin and 161 (IQR: 100–244; range: 63–750) for beta-defensin. Secretory IgA concentrations below the reference range had a median of 125.5 μg/g (IQR: 85.0–251.5 μg/g; range: 45–509 μg/g), whereas concentrations above the reference range had a median of 4032.5 μg/g (IQR: 2871.5–5437.0 μg/g; range: 2081–7800 μg/g).

### 3.3. Distribution of Dysbiosis Domains According to Sex and Place of Residence

When stratified by sex, core bacterial imbalance was significantly more frequent in women than in men (96.2% vs. 80.0%, *p* = 0.021). No significant sex-related differences were observed for protective bacterial depletion, opportunistic bacterial overgrowth, fungal overgrowth, or functional and mucosal immune abnormalities (all *p* > 0.05) (Table 3).

Similarly, no significant differences were identified between urban and rural participants regarding any of the five predefined dysbiosis domains (all *p* > 0.05) (Table 4).

### 3.4. Integrated Dysbiosis Pattern Profile

**Figure 2:**
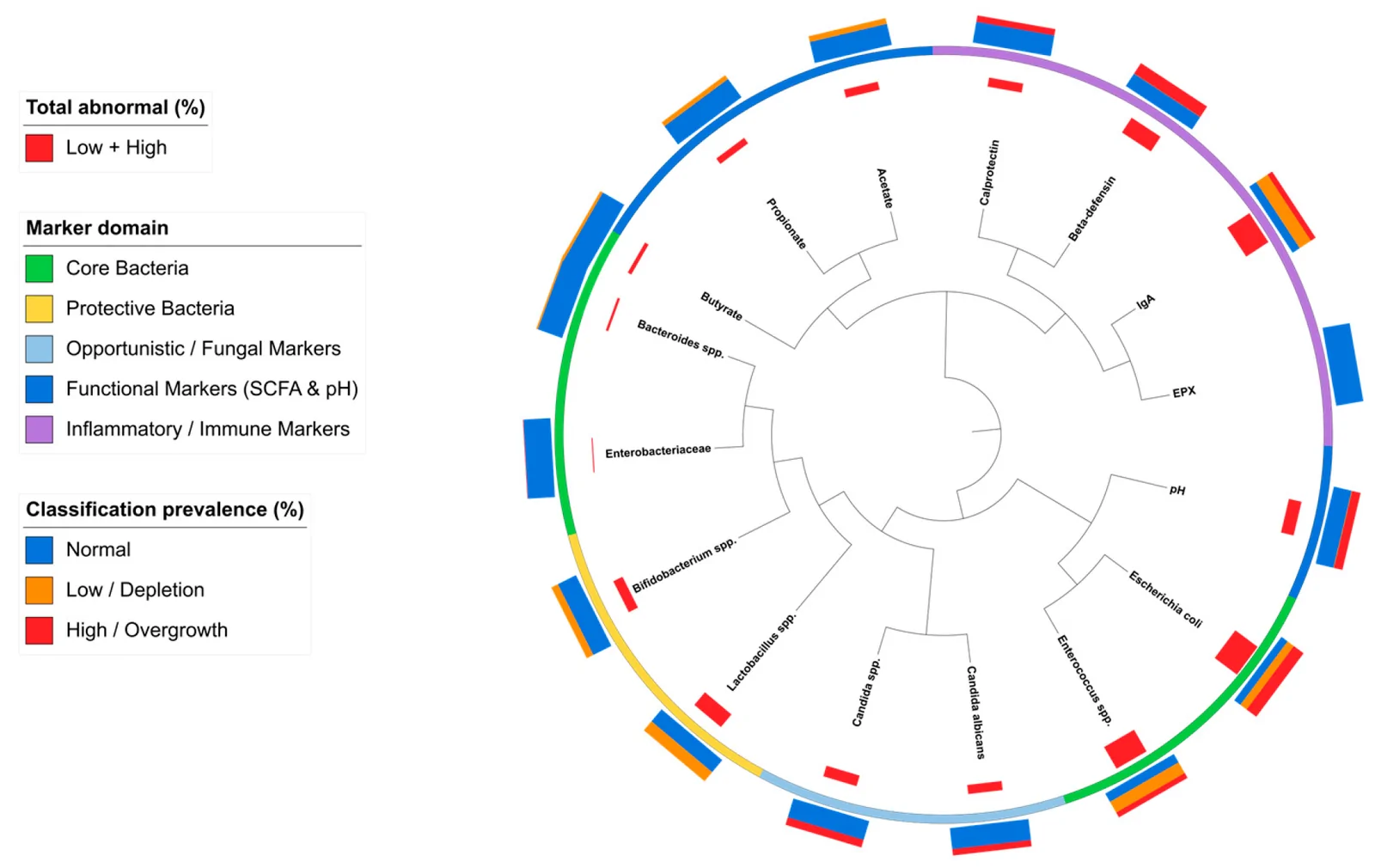
Integrated   microbiota dysbiosis signature. Circular   visualization of microbiota-related laboratory markers grouped into core bacterial, protective bacterial, opportunistic/fungal, functional, and inflammatory/immune domains. Outer tracks indicate the proportions of normal and abnormal results according to laboratory reference intervals. The figure represents a graphical summary of dysbiosis-related laboratory patterns and is not a phylogenetic tree.

Based on the predefined dysbiosis domains, at least one microbiota-related abnormality was identified in 97 (99.0%) of 98 participants. The most prevalent domains were core bacterial imbalance, detected in 87 (88.8%) participants, and inflammatory or immune abnormalities, detected in 85 (86.7%) participants. Opportunistic bacterial overgrowth was observed in 61 (62.2%) participants, protective bacterial depletion in 58 (59.2%), and fungal overgrowth in 49 (50.0%). Functional abnormalities included low SCFA markers in 27 (27.6%) participants and abnormal fecal pH in 33 (33.7%) participants. According to dysbiosis complexity, 7 (7.1%) participants exhibited mild dysbiosis, 36 (36.7%) moderate dysbiosis, and 54 (55.1%) complex dysbiosis involving three or more altered domains. Overall, dysbiosis-related abnormalities most frequently occurred as overlapping multi-domain patterns rather than isolated findings (Figure 2).

### 3.5. Correlation Structure Between Microbiota, SCFA, pH, and Immune Markers

**Figure 3:**
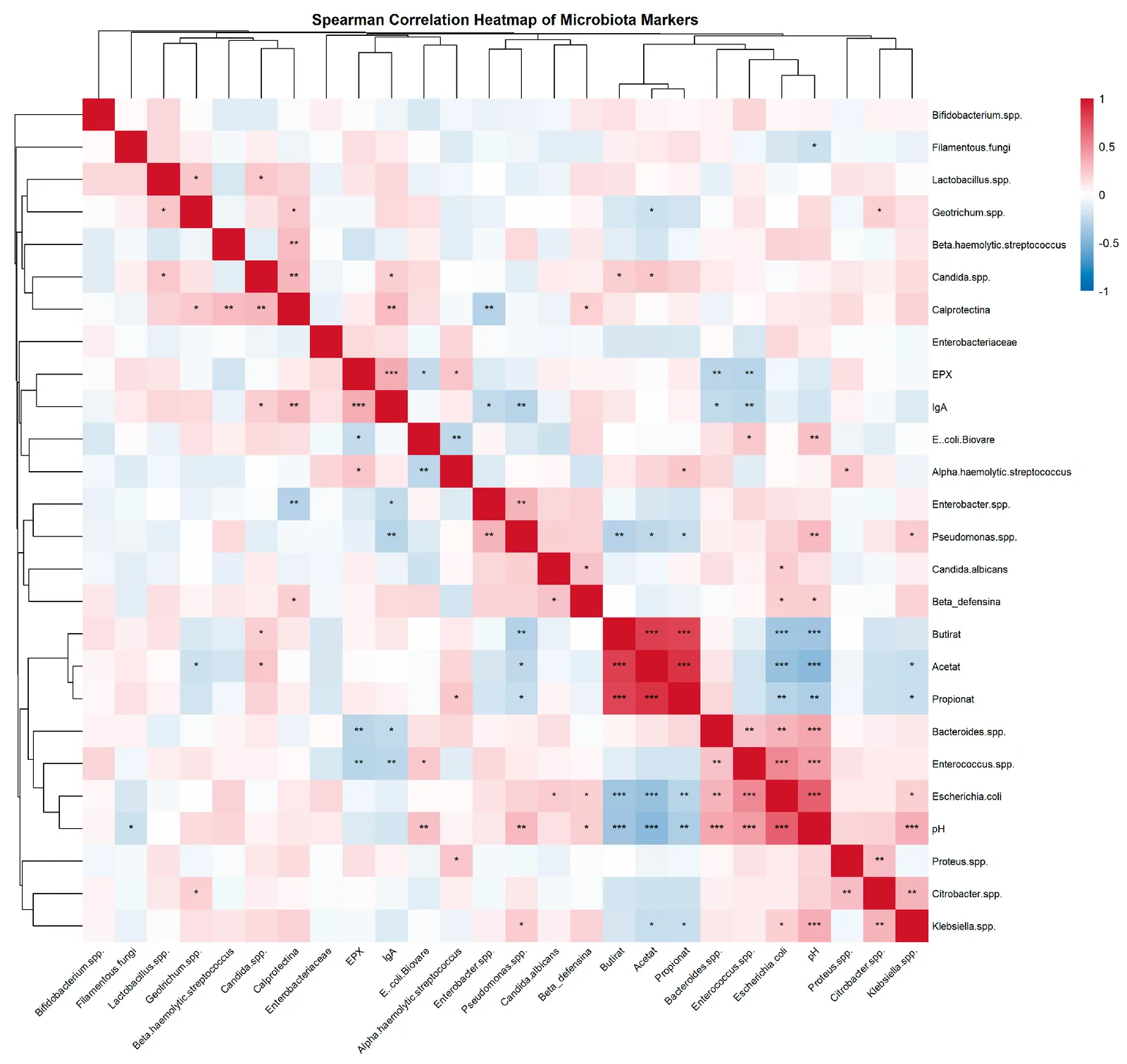
Spearman correlation heatmap of microbiota, SCFA markers, fecal pH, and mucosal immune markers. Red indicates positive correlations, blue indicates negative correlations, and white indicates weak or absent correlations. Hierarchical clustering was performed using average linkage based on the similarity of marker correlation profiles. Statistical significance is indicated as * *p* < 0.05, ** *p* < 0.01, and *** *p* < 0.001.

Spearman correlation analysis was performed to evaluate associations between microbiota-related markers, SCFA markers, fecal pH, and mucosal immune parameters (Figure 3). The strongest positive correlations were observed among the SCFA markers acetate, propionate, and butyrate, which clustered together in the hierarchical analysis. Fecal pH showed inverse correlations with several SCFA markers. Additional associations were observed between microbiota-related and immune markers, including inverse correlations between *Bacteroides* spp. and selected mucosal immune parameters. Immune markers, including calprotectin, EPX, beta-defensin, and secretory IgA, also demonstrated clustering patterns within the correlation matrix.

## 4. Discussion

### 4.1. Principal Findings and Relationship with the Microbiota–Human Health Axis

This laboratory-based observational cross-sectional study evaluated microbiota-related, metabolic, fungal, and mucosal immune abnormalities in asymptomatic adults undergoing stool microbiota screening. The main finding was the high prevalence of laboratory-defined dysbiosis-related abnormalities. At least one microbiota-related abnormality was identified in 97 of 98 participants (99.0%), and complex dysbiosis involving three or more altered biological domains was present in 54 participants (55.1%). The most frequent broad dysbiosis domains were core bacterial imbalance, inflammatory or mucosal immune marker abnormalities, opportunistic bacterial overgrowth, protective bacterial depletion, and fungal overgrowth.

These findings should be interpreted within the broader microbiota–human health framework. John et al. emphasized that the intestinal microbiota participates in metabolic regulation, immune homeostasis, epithelial barrier maintenance, inflammatory control, and systemic host physiology [23,24,25,26]. In this context, the present study extends the microbiota–human health discussion from disease-centered cohorts toward an asymptomatic screening population, showing that microbiota-related laboratory abnormalities may be detectable even in the absence of acute gastrointestinal symptoms.

The high prevalence of dysbiosis-related abnormalities supports the concept that dysbiosis should not be interpreted as a single abnormal microorganism or as a simple binary normal/abnormal state. Petersen and Round described dysbiosis as a disturbance that can influence host immunity and disease susceptibility, while Hooks and O’Malley emphasized the conceptual complexity and limitations of using dysbiosis as a rigid diagnostic label [27,28]. Therefore, the present study interpreted dysbiosis as a multidimensional laboratory phenotype involving microbial composition, protective commensal depletion, opportunistic expansion, fungal overgrowth, microbial metabolic activity, fecal pH, and mucosal immune response.

The finding that 99.0% of participants had at least one abnormal laboratory marker should not be interpreted as evidence that almost all participants had gastrointestinal disease. Population-level studies have shown that the healthy gut microbiome is highly individualized and strongly influenced by diet, age, medication exposure, host factors, geography, environmental exposure, body mass index, and lifestyle [18,29,30,31,32]. Therefore, isolated deviations from laboratory reference intervals may be common in screening contexts. The more biologically meaningful observation in the present study is not the presence of any single abnormality, but the frequent coexistence of abnormalities across multiple domains.

### 4.2. Comparison with Asymptomatic, Healthy, and Screening-Based Microbiome Studies

The findings of the present study are most appropriately interpreted in the context of studies performed in healthy, asymptomatic, non-diseased, or screening-based adult populations, rather than disease-centered cohorts. Large reference studies of healthy adults have shown that the gut microbiome varies substantially between individuals and that a single universal “normal” microbiome profile cannot be defined. The Human Microbiome Project demonstrated marked interindividual variability in microbial community structure among healthy adults, while Lloyd-Price et al. emphasized that the healthy human microbiome is better understood as a range of ecological configurations compatible with health rather than as one fixed microbial composition [18,22].

This concept is directly relevant to the high prevalence of laboratory-defined abnormalities observed in the present cohort. Almonacid et al. analyzed stool samples from 897 healthy individuals and proposed 16S rRNA-based healthy reference ranges for clinically relevant gut microbial taxa [29]. Similarly, Watanabe et al., in the Mykinso Cohort Study, established reference ranges for gut microbial indices in a real-world Japanese population and reported substantial interindividual variability, with no single “typical” gut microbiota profile [30]. Compared with these healthy-reference studies, the present analysis used laboratory reference intervals rather than sequencing-derived abundance profiles. Nevertheless, the overall interpretation is similar: microbiota-related markers in asymptomatic adults should be interpreted as distributed biological variation rather than as a rigid binary distinction between normal and pathological states.

Population-based studies further support this interpretation. Falony et al. showed in the Flemish Gut Flora Project that stool consistency, medication exposure, diet-related variables, and host factors are associated with gut microbiota composition [31]. Zhernakova et al. similarly reported in the LifeLines-DEEP cohort that diet, medication use, blood parameters, and host-related factors were associated with microbiome composition and diversity [32]. These findings are important for interpreting the present cohort because detailed diet, medication history beyond exclusion windows, lifestyle, body mass index, and metabolic variables were not systematically modeled. Therefore, some of the marker-level abnormalities observed in this study may reflect unmeasured host and lifestyle-related variability rather than disease-specific dysbiosis.

A particularly relevant comparison comes from screening-based populations. Hua et al. analyzed the gut microbiome using shotgun metagenomic sequencing in adults undergoing screening colonoscopy and emphasized the importance of demographic, lifestyle, medication-related, body mass index, and dietary variables when interpreting microbiome differences in asymptomatic individuals [33]. In the present study, medication- and procedure-related exposures were recorded only for eligibility screening and exclusion purposes, including recent antibiotic use, proton pump inhibitor use, acute diarrheal symptoms, active hemorrhoidal bleeding, and menstruation at sampling. These exclusion criteria were used to reduce pre-analytical and clinical confounding before inclusion in the final cohort. However, detailed dietary intake, body mass index, physical activity, lifestyle variables, comorbidities, and medication history beyond the predefined exclusion criteria were not systematically collected and could not be incorporated into multivariable analyses.

Overall, these comparisons should be interpreted with caution because the present study used a culture-based and laboratory biomarker-based approach rather than sequencing-based microbiome profiling. Sequencing-based studies primarily describe relative microbial abundance, diversity, taxonomic composition, and, in some cases, metagenomic functional potential. In contrast, the present study classified selected bacterial and fungal groups, SCFA markers, fecal pH, and mucosal immune biomarkers according to laboratory-defined reference intervals. Therefore, the findings are not directly interchangeable with sequencing-derived microbiome profiles. The strength of the present approach is that it integrates microbial, fungal, metabolic, luminal, and mucosal immune domains in a clinically interpretable laboratory framework. However, differences in analytical method, taxonomic resolution, reporting format, reference intervals, and population characteristics limit direct numerical comparison with previously published sequencing-based cohorts.

### 4.3. Marker-Level Abnormalities in Relation to Asymptomatic Reference Populations

At the marker level, the most frequent abnormalities in the present cohort included low secretory IgA, low *Enterococcus* spp., increased beta-defensin, increased *Escherichia coli*, low *Lactobacillus* spp., increased fecal pH, increased *Candida* spp., low *Bifidobacterium* spp., increased *Candida albicans*, and increased calprotectin. Several large studies have established microbiota reference patterns in healthy populations using sequencing-based approaches [29,30]. While methodological differences preclude direct comparison, these studies provide an important context for interpreting the present findings.

Almonacid et al. analyzed stool samples from 897 self-reported healthy individuals and proposed healthy reference ranges for 28 clinically relevant gut microbial taxa using 16S rRNA gene sequencing [29]. Watanabe et al. analyzed 5843 Japanese individuals from the Mykinso Cohort Study and established gut microbial reference ranges after accounting for epidemiological variables, reporting high beta-diversity and no single “typical” gut microbiota profile [30]. Compared with these healthy-reference studies, the present cohort showed a high frequency of marker-level deviations, but these deviations should not be interpreted as disease-specific signatures. Rather, they indicate that when multiple microbiota-related, fungal, metabolic, pH, and mucosal immune markers are assessed simultaneously, deviations from laboratory reference intervals may be common even in asymptomatic adults.

Low *Bifidobacterium* spp. was observed in 26.5% of participants in the present study. In healthy adults, *Bifidobacterium* is commonly detected but varies markedly between individuals; the previous literature reports that adult bifidobacterial levels may generally fall within approximately 2–14% relative abundance, depending on age, diet, geography, and analytical method [34]. Therefore, the 26.5% frequency of low *Bifidobacterium* spp. in the present cohort suggests reduced protective bacterial representation in a subset of asymptomatic adults. Nevertheless, reduced *Bifidobacterium* levels may contribute to a less resilient microbial ecosystem and could represent one component of the broader dysbiosis pattern identified in this cohort.

Low *Lactobacillus* spp. was detected in 42.9% of participants. Published culture-based healthy-population comparators are limited, but Prete et al. analyzed viable fecal bacteria in healthy adult volunteers using selective culture media and reported mesophilic lactobacilli counts of 7.78 ± 0.54 log10 CFU/g in omnivores, 7.00 ± 0.97 log10 CFU/g in lacto-ovo-vegetarians, and 7.16 ± 0.75 log10 CFU/g in vegans [35]. Compared with these culture-based healthy-volunteer data, the present finding of low *Lactobacillus* spp. in 42.9% of asymptomatic adults suggests that reduced lactobacilli were relatively frequent in our screening cohort. However, direct comparison remains limited because Prete et al. reported group-level viable counts, whereas the present study classified individual results as low or normal according to laboratory reference intervals. Biologically, this finding remains relevant because *Lactobacillus* spp. contributes to colonization resistance, epithelial barrier support, local pH modulation, and interaction with mucosal immune responses [5,6,7,27].

Jendraszak et al. also provide a relevant methodological comparison because they analyzed adult stool samples in a commercial diagnostic microbiota-testing context and assessed selected gut microorganisms together with fecal biomarkers, including calprotectin and secretory IgA [36]. Their study reported that most biomarker concentrations remained within reference ranges and highlighted the importance of participant-level factors such as age, BMI, probiotic use, and self-reported health status when interpreting stool microbiota and biomarker results. Compared with that study, the present analysis similarly used a laboratory-screening framework, but it focused specifically on asymptomatic adults and did not systematically collect BMI, dietary, lifestyle, or detailed medication variables. Therefore, although culture-based and diagnostic-laboratory comparators exist, methodological and covariate differences still limit direct one-to-one comparison.

Increased *E. coli* was detected in 43.9% of participants. As a common member of the facultative gut microbiota, *E. coli* may be present in healthy individuals and does not necessarily indicate infection or pathogenicity. Therefore, the observed increase may reflect expansion of facultative bacterial populations within a broader dysbiosis pattern. Similar taxa have been reported across healthy populations, although substantial interindividual variability exists [29,30]. However, because strain-level characterization was not performed, it was not possible to distinguish commensal strains from potentially pro-inflammatory or pathogenic variants.

This distinction is important because taxonomic identification at the genus or species level does not necessarily indicate biological behavior. Within the same bacterial group, some strains may behave as harmless commensals, whereas others may carry virulence-associated traits, show greater inflammatory potential, or expand under conditions of reduced colonization resistance. Therefore, increased abundance of facultative organisms in the present study should be interpreted as a marker of altered microbial ecology rather than direct evidence of pathogenic colonization. Future studies using sequencing-based, metagenomic, or strain-resolved approaches would be required to determine whether these culture-based abnormalities reflect benign compositional variation or expansion of functionally relevant pro-inflammatory strains.

*Bacteroides* spp. were mostly preserved, with low values observed in only 6.1% of participants. This finding suggests that a generalized loss of anaerobic bacterial markers did not characterize the dysbiosis profile in the present asymptomatic cohort. In healthy adults, *Bacteroides*-related taxa commonly represent an important component of the intestinal microbiota, although their relative abundance varies substantially between individuals and populations [29,30]. Therefore, the low frequency of *Bacteroides* spp. depletion in our cohort suggests a selective pattern of dysbiosis, mainly involving protective bacterial depletion, facultative bacterial imbalance, fungal overgrowth, altered fecal pH, functional metabolic changes, and mucosal immune marker abnormalities.

Increased *Candida* spp. was observed in 28.6% of participants, and increased *Candida albicans* in 23.5%. These findings should be interpreted cautiously because intestinal carriage and overgrowth are not equivalent. Delavy et al. detected *Candida albicans* carriage in 82.9% of healthy individuals using quantitative PCR [37], indicating that the presence of Candida is common in asymptomatic populations. Therefore, increased Candida markers in the present cohort are more likely to reflect alterations in bacterial–fungal ecological balance than clinically significant fungal disease, particularly when accompanied by protective bacterial depletion, altered fecal pH, or mucosal immune marker abnormalities. The relatively high frequency of Candida markers may also reflect population-specific factors. Previous widespread antibiotic exposure, which has been reported in Romania and other Eastern European countries, may contribute to disruption of bacterial–fungal ecological balance and favor fungal expansion. However, antibiotic exposure was not evaluated in the present study.

Fecal pH was increased in 31.6% of participants, while low SCFA markers were less frequent (acetate in 21.4%, propionate in 17.3%, and butyrate in 10.2%). These findings suggest that functional metabolic dysbiosis affected only a subset of the cohort. In healthy populations, microbial metabolic activity is influenced by dietary patterns, fiber intake, stool characteristics, microbiota composition, and host-related factors [31,32]. The coexistence of increased fecal pH and reduced SCFA levels in part of the cohort may therefore reflect alterations in microbial fermentation and metabolic activity within the intestinal ecosystem.

In healthy adults, fecal calprotectin concentrations are generally low and typically remain below the clinical threshold of 50 μg/g; for example, Park et al. reported a mean fecal calprotectin concentration of approximately 15.88 μg/g in adults younger than 50 years of age [38]. The detection of elevated fecal calprotectin in a subset of our asymptomatic participants may reflect low-grade mucosal inflammatory activity and is consistent with the concomitant microbiota and mucosal immune alterations observed in this cohort. However, because all participants were asymptomatic and no endoscopic or histological assessment was performed, elevated fecal calprotectin should be interpreted as a nonspecific laboratory biomarker rather than diagnostic evidence of inflammatory bowel disease. These findings suggest that a subset of apparently healthy adults may exhibit early alterations in intestinal homeostasis that warrant longitudinal follow-up to determine their persistence and potential clinical significance.

Secretory IgA abnormalities were frequent in this cohort, with both decreased and increased concentrations observed. Direct comparison with healthy asymptomatic populations remains challenging because fecal secretory IgA is not routinely included in large population-based microbiome studies. Secretory IgA plays a central role in maintaining intestinal immune homeostasis by regulating host–microbiota interactions and limiting microbial adherence to the intestinal epithelium. Reduced secretory IgA may reflect impaired mucosal immune defense, whereas increased concentrations may indicate enhanced mucosal immune activation in response to microbial or environmental stimuli. Therefore, isolated alterations in secretory IgA should not be interpreted as evidence of gastrointestinal disease but rather as indicators of altered mucosal immune function. In the present study, the coexistence of secretory IgA abnormalities with bacterial, fungal, functional, and inflammatory marker alterations supports the presence of disturbed host–microbiota homeostasis in a subset of otherwise asymptomatic adults. Longitudinal studies are warranted to determine whether these immune alterations are transient or represent persistent changes associated with future gastrointestinal or systemic disease risk.

Beta-defensin is a key component of the intestinal innate immune system and is produced by epithelial cells in response to microbial stimulation. Increased fecal beta-defensin may therefore reflect activation of the mucosal epithelial defense rather than disease-specific inflammation. However, comparable reference data from large cohorts of healthy asymptomatic adults remain limited, making direct comparisons difficult. In the present study, the coexistence of increased beta-defensin with alterations in fecal calprotectin, secretory IgA, bacterial composition, fungal markers, and functional biomarkers suggests a coordinated mucosal immune response associated with disturbed host–microbiota homeostasis.

Overall, these findings support the concept that microbiota-related alterations are better interpreted as an integrated laboratory pattern than as isolated biomarker abnormalities. Although the clinical implications remain unclear, the combined abnormalities observed across microbial, functional, and mucosal immune markers suggest reduced resilience of the intestinal ecosystem. Future longitudinal studies are needed to determine whether these alterations represent transient biological variability or persistent changes associated with later clinical outcomes.

### 4.4. Integrated Dysbiosis Domains and Complexity

The domain-based analysis showed that core bacterial imbalance was the most prevalent dysbiosis domain, detected in 88.8% of participants. Inflammatory or mucosal immune marker abnormalities were detected in 86.7%, opportunistic bacterial overgrowth in 62.2%, protective bacterial depletion in 59.2%, and fungal overgrowth in 50.0%. Functional abnormalities were also observed, including low SCFA markers in 27.6% and abnormal fecal pH in 33.7%.

This multi-domain structure supports the interpretation of dysbiosis as an ecosystem-level disturbance rather than an isolated taxonomic abnormality. Petersen and Round emphasized the interaction between dysbiosis, host immunity, and disease susceptibility, while Hooks and O’Malley highlighted the need for cautious and context-dependent use of the term dysbiosis [27,28]. The present findings align with this view because the abnormalities were distributed across bacterial, fungal, metabolic, luminal, and mucosal immune domains.

More than half of the participants showed complex dysbiosis, defined as three or more altered domains. This finding suggests that in asymptomatic microbiota screening, the most relevant signal may be the accumulation of abnormalities across domains rather than the presence of a single abnormal marker. Nevertheless, “complex dysbiosis” should not be interpreted as clinical severity. It reflects the number of altered laboratory domains, not symptom burden, inflammatory activity, endoscopic disease, or prognosis. This distinction is especially important because all participants were asymptomatic at the time of stool sample collection.

### 4.5. Sex-Related and Urban–Rural Comparisons

When dysbiosis domains were stratified according to sex, core bacterial imbalance was significantly more frequent in women than in men. No significant sex-related differences were observed for protective bacterial depletion, opportunistic bacterial overgrowth, fungal overgrowth, or functional and mucosal immune abnormalities. This suggests that the sex-related signal was limited mainly to the core bacterial domain rather than reflecting a global difference across all dysbiosis categories.

Previous studies have reported that sex and sex hormones may influence gut microbiota composition, although human data remain heterogeneous and may be confounded by diet, body mass index, age, medication exposure, lifestyle, and hormonal status [39,40,41]. Kim et al. reviewed sex differences in gut microbiota, while Yoon and Kim discussed the role of sex hormones and gender-related factors in shaping gut microbial communities [39,40]. Hatayama et al. also reported sex-associated differences in intestinal microbiota and their relationship with host factors in a population-based setting [41]. Therefore, the significant sex-related difference observed in this study is biologically plausible, but it should be interpreted cautiously because the study was not specifically designed to evaluate sex-specific microbiota mechanisms.

No significant differences were identified between urban and rural participants for the predefined dysbiosis domains. This finding contrasts with population-level studies showing that geography, migration, environmental exposures, and lifestyle can strongly influence microbiome structure [31,32]. He et al. analyzed gut microbiome variation across geographically distinct healthy populations and showed that healthy microbiome reference ranges and disease-associated microbiome models may not be universally transferable across regions, because baseline microbiome composition differs substantially between populations [31]. This finding is clinically and biologically relevant because it suggests that microbiota results should be interpreted in the context of regional background variation rather than against a single universal “healthy” profile. Vangay et al. studied immigrant and refugee populations and found that migration from a non-Western country to the United States was associated with rapid loss of gut microbiome diversity and function, with progressive replacement of native microbial strains and functions by U.S.-associated microbiome features [32]. This supports the concept that westernization, diet, and environmental transition can reshape the gut microbiome over time. In the present study, the absence of significant urban–rural differences may reflect limited sample size, incomplete environmental exposure characterization, outpatient screening selection, or the use of laboratory marker-based testing rather than sequencing-based microbiome profiling. Therefore, this negative finding should not be interpreted as evidence that environment has no influence on microbiota composition.

### 4.6. Functional Dysbiosis: SCFA and Fecal pH

Functional abnormalities were present in a subset of participants. Low acetate was detected in 21.4%, low propionate in 17.3%, and low butyrate in 10.2%, while abnormal fecal pH was present in 33.7%. In the Spearman correlation heatmap, acetate, propionate, and butyrate clustered together and showed strong positive intercorrelations, supporting their interpretation as a shared functional metabolic axis.

These findings are consistent with the biological role of SCFAs as major microbial fermentation products. SCFAs contribute to epithelial barrier integrity, colonocyte energy metabolism, mucosal immune regulation, and anti-inflammatory signaling [5,42,43]. Mann et al. described acetate, propionate, and butyrate as microbiota-derived metabolites whose intestinal and systemic availability is shaped by environmental factors such as diet and antibiotic exposure. They emphasized that SCFAs can regulate immune responses locally in the gut and at extra-intestinal sites, including effects relevant to infection, intestinal inflammation, autoimmunity, allergy, asthma, and responses to cancer therapy [42]. Nogal et al. further summarized that resistant dietary carbohydrates are fermented by gut bacteria into SCFAs and that these metabolites may influence cardiometabolic health through mechanisms involving appetite regulation, glucose homeostasis, lipid metabolism, blood pressure regulation, gut barrier function, and inflammatory signaling [43]. Therefore, reduced SCFA markers in the present study may indicate impaired microbial fermentation capacity and represent a functional component of dysbiosis.

The inverse relationship between fecal pH and SCFA markers observed in the heatmap is biologically plausible. SCFAs contribute to luminal acidification; therefore, reduced SCFA availability may be associated with a less acidic fecal environment. Increased fecal pH, may reflect altered fermentation dynamics and may influence ecological conditions that favor opportunistic expansion. However, fecal SCFA concentrations are influenced by microbial production, host absorption, diet, fiber intake, intestinal transit time, and stool water content.

### 4.7. Fungal Overgrowth and Bacterial–Fungal Dysbiosis

Fungal overgrowth was identified in 50.0% of participants, with increased *Candida* spp. and *Candida albicans* representing the main fungal abnormalities. Increased *Candida* spp. was detected in 28.6%, while increased *Candida albicans* was detected in 23.5%. These findings indicate that fungal markers contributed substantially to the laboratory-defined dysbiosis profile.

The intestinal mycobiota is increasingly recognized as an important component of gut ecosystem homeostasis. Previous studies and reviews have shown that fungal dysbiosis, particularly involving Candida species, may interact with bacterial communities and mucosal immune pathways in inflammatory gastrointestinal disorders [9,10,11,44,45]. Li et al. showed that fungal strain diversity can shape host immune responses in inflammatory bowel disease, indicating that fungi are not only passive colonizers but may actively influence intestinal inflammatory pathways [44]. Their findings support the biological relevance of fungal strain-level variation and suggest that different fungal strains may have distinct immunologic effects. Underhill and Braun further discussed that the intestinal fungal microbiome can interact with bacterial communities and mucosal immune pathways, and that altered fungal communities may contribute to intestinal inflammation through mechanisms involving epithelial barrier interaction, innate immune recognition, and host inflammatory signaling [45]. These studies are clinically and biologically relevant to the present findings because they support the interpretation of increased *Candida* markers as part of a broader bacterial–fungal–immune dysbiosis pattern rather than as an isolated fungal abnormality.

The association between protective bacterial depletion and fungal overgrowth is biologically plausible. *Lactobacillus* spp. may restrict fungal expansion through competition for adhesion sites and nutrients, production of antimicrobial metabolites, modulation of local pH, and interaction with mucosal immunity [6,7,9]. Therefore, increased Candida markers in this study should not be interpreted as invasive fungal disease or necessarily as symptomatic candidiasis. Instead, they should be interpreted as laboratory markers of altered intestinal ecological balance requiring clinical correlation and future validation using dedicated mycobiome analysis.

### 4.8. Mucosal Immune and Inflammatory Marker Abnormalities

Mucosal immune and inflammatory marker abnormalities were frequent. Increased beta-defensin was observed in 44.9% of participants, increased calprotectin in 23.5%, low secretory IgA in 49.0%, and high secretory IgA in 20.4%. EPX remained within the reference range in all participants. These findings suggest that dysbiosis-related laboratory patterns were accompanied by measurable mucosal immune marker changes.

This observation is consistent with the established concept that the gut microbiota and mucosal immune system are closely interconnected [4,6,13,46,47]. Secretory IgA plays a central role in immune exclusion, microbial containment, and regulation of host–microbiota interactions [46,47]. Mantis et al. described the complex roles of secretory IgA in mucosal immunity and gut homeostasis, while Pietrzak et al. emphasized secretory IgA as an adaptive barrier against microbial cells [46,47]. Therefore, the high frequency of low secretory IgA in this cohort may suggest altered mucosal immune containment or impaired host–microbiota interaction. However, secretory IgA may also be influenced by stress, diet, infections, exercise, immune status, and mucosal exposure history, so isolated abnormalities cannot be interpreted as disease specific.

Increased beta-defensin may reflect activation of innate antimicrobial epithelial responses. Enteric defensins are involved in maintaining mucosal barrier defense and shaping microbiota composition [48]. Therefore, the high prevalence of increased beta-defensin may indicate epithelial antimicrobial response to altered microbial ecology. Increased calprotectin was observed in 23.5% of participants. Calprotectin is widely used as a non-invasive marker of intestinal inflammatory activity, particularly in inflammatory bowel disease, but mild elevations may also occur in other contexts and require clinical interpretation [49,50]. Jukic et al. discussed calprotectin not only as a biomarker but also as a molecule with biological function in inflammation, while Ricciuto and Griffiths reviewed its clinical value in intestinal inflammation [50,51].

The immune-marker findings may also be interpreted in relation to broader models of microbial exposure and immune dysregulation. Previous work has highlighted how prolonged host–microbial interactions may contribute to persistent immune activation and inflammatory signaling beyond the initial triggering event [52]. Although the mechanisms differ across conditions, these observations support the broader concept that microbial and immune pathways remain closely interconnected. In the present cohort, the alterations in secretory IgA, beta-defensin, and calprotectin should therefore be interpreted as mucosal immune signals associated with dysbiosis-related laboratory patterns, not as proof of systemic immune-mediated disease.

The normal EPX profile suggests that eosinophil-associated mucosal activity was not a dominant feature in this cohort, despite abnormalities in other immune markers. This distinction strengthens the interpretation that immune marker changes were not uniform across all inflammatory pathways, but rather involved selected mucosal immune and epithelial response markers.

### 4.9. Interpretation of the Circular Dysbiosis Signature and Spearman Heatmap

The circular dysbiosis signature and Spearman correlation heatmap provide complementary visual summaries of the study findings. The circular figure showed that abnormalities were distributed across core bacterial, protective bacterial, opportunistic/fungal, functional metabolic, and inflammatory/mucosal immune domains. This visualization supports the interpretation that dysbiosis-related abnormalities were structured across multiple biological systems rather than randomly distributed.

The Spearman heatmap provided additional support for this integrated interpretation. The strongest and most biologically coherent correlation structure was observed among acetate, propionate, and butyrate. This finding is consistent with their shared origin as microbial fermentation products and with the previous literature describing SCFAs as a functional link between diet, gut microbiota, epithelial barrier integrity, and immune regulation [5,42,47]. The inverse relationship between fecal pH and SCFA markers further supports the biological plausibility of the functional marker profile.

The immune marker correlations were more heterogeneous, which is expected given the complexity of host–microbiota immune interactions. Secretory IgA, beta-defensin, calprotectin, and EPX reflect different aspects of mucosal immunity, including immune exclusion, epithelial antimicrobial response, neutrophil-associated inflammation, and eosinophil-associated activity [46,47,48,49,50]. Therefore, the heatmap should be interpreted as an exploratory representation of microbial–metabolic–immune associations rather than as evidence of causal pathways.

Both visualizations have methodological limitations. The circular dendrogram-style figure is not a sequencing-derived phylogenetic tree and does not represent evolutionary or taxonomic distance. It is a graphical organization of laboratory marker domains and prevalence patterns. Similarly, the heatmap is based on cross-sectional correlations and cannot establish directionality, causality, or temporal sequence.

### 4.10. Clinical and Preventive Implications

The findings have important implications for the interpretation of microbiota screening in asymptomatic adults. A key message of this study is that isolated laboratory abnormalities are common and should not automatically be equated with disease. This interpretation is supported by studies showing that the healthy microbiome is highly individualized and lacks a universal reference profile applicable to all populations [18,22,29,30,31,32].

The clinically more meaningful signal may be the presence of multi-domain patterns, particularly when protective bacterial depletion, opportunistic or fungal overgrowth, reduced SCFA markers, abnormal fecal pH, and mucosal immune abnormalities occur together. Such patterns may better reflect impaired gut ecosystem stability than isolated marker deviations. This supports a pattern-based approach to microbiota interpretation.

From a preventive perspective, the findings justify further investigation of modifiable factors that influence microbiota resilience, including diet, fiber intake, medication exposure, antibiotic history, proton pump inhibitor use, metabolic status, sleep, stress, physical activity, and environmental exposures [8,29,30,31,32,42,43]. However, the present study does not demonstrate that microbiota-directed interventions improve outcomes in asymptomatic individuals. Therefore, therapeutic recommendations cannot be made from these results alone. From a clinical perspective, laboratory findings related to gut dysbiosis should always be interpreted in the context of the individual patient, as factors such as age, symptoms, nutritional and metabolic status, medication use, and lifestyle may influence the gut microbiota. In our study, all participants were asymptomatic, and demographic data included age, sex, and place of residence. Accordingly, the findings reflect microbiota and biomarker patterns observed in an apparently healthy population and should be interpreted within this context.

### 4.11. Strengths and Limitations

Several limitations should be acknowledged. First, the cross-sectional design does not allow assessment of temporal or causal relationships. Therefore, the findings describe laboratory-defined intestinal ecosystem patterns observed in asymptomatic adults at the time of assessment.

Second, the study used a culture- and biomarker-based approach rather than sequencing-based microbiome profiling. While this allowed integrated assessment of bacterial, fungal, metabolic, luminal, and mucosal immune markers, strain-level characterization was not available, limiting differentiation between potentially distinct variants within the same bacterial group [21,22].

Finally, participant-level analyses were based on age, sex, and place of residence, while additional clinical, anthropometric, dietary, and lifestyle variables were not systematically collected. Longitudinal follow-up was not part of the study design. Given the absence of a universally accepted laboratory definition of dysbiosis, particularly for culture-based testing [27,28], the findings should be interpreted within the predefined laboratory framework used in this study.

### 4.12. Future Research Directions

Future studies should use longitudinal designs to determine whether subclinical dysbiosis-related laboratory abnormalities persist over time, normalize spontaneously, or predict the later development of gastrointestinal, metabolic, inflammatory, or immune-mediated conditions. Repeated stool testing would help clarify the temporal stability and clinical relevance of the patterns observed in this study.

Sequencing-based methods, including 16S rRNA sequencing, shotgun metagenomics, metabolomics, and dedicated mycobiome profiling, should be incorporated in future studies. These approaches would allow more precise characterization of bacterial and fungal communities, microbial diversity, strain-level features, functional pathways, and metabolic potential [21,22,44,45]. Integration of SCFA profiling, fecal pH, and mucosal immune biomarkers may also help distinguish functional dysbiosis from isolated compositional variation [42,43,44,45,46,47,48,49,50].

Future research should also evaluate composite dysbiosis scores integrating protective bacterial depletion, opportunistic bacterial expansion, fungal overgrowth, SCFA markers, fecal pH, beta-defensin, calprotectin, secretory IgA, and additional mucosal immune markers. Such models may help distinguish clinically meaningful dysbiosis signatures from minor interindividual microbiota variation. Interventional studies will be required to determine whether dietary, prebiotic, probiotic, or lifestyle-based interventions can modify these signatures and improve clinically relevant outcomes.

## 5. Conclusions

In the present study, dysbiosis-related laboratory abnormalities were highly prevalent among asymptomatic adults undergoing microbiota screening. Most participants exhibited abnormalities involving multiple biological domains, including bacterial imbalance, protective bacterial depletion, opportunistic or fungal overgrowth, functional metabolic alterations, fecal pH disturbances, and mucosal immune marker abnormalities. These findings suggest that dysbiosis in asymptomatic individuals may be better characterized as a multidimensional laboratory phenotype rather than as an isolated microbial abnormality.

The findings should be interpreted as prevalence and pattern data rather than evidence of gastrointestinal disease or future disease risk. Although the observed abnormalities frequently co-occurred across microbial, metabolic, and immune domains, the cross-sectional design does not permit causal or prognostic conclusions. Longitudinal studies incorporating sequencing-based microbiome profiling, mycobiome analysis, metabolomics, and clinical follow-up are needed to clarify the biological and clinical significance of laboratory-defined dysbiosis in asymptomatic adults.

## Figures and Tables

**Table 1:** Laboratory parameters and classification criteria.

Parameter	Reference Interval	Classification Used in Analysis
*Enterococcus* spp.	10^6^–10^8^ CFU/g	Low/Normal/High
*Escherichia coli*	10^6^–10^8^ CFU/g	Low/Normal/High
*Bacteroides* spp.	≥10^8^ CFU/g	Low/Normal
*Bifidobacterium* spp.	≥10^8^ CFU/g	Low/Normal
*Lactobacillus* spp.	≥10^5^ CFU/g	Low/Normal
*Enterobacteriaceae*	≤10^6^ CFU/g	Normal/High
*Candida* spp.	≤10^3^ CFU/g	Normal/High
*Candida albicans*	≤10^3^ CFU/g	Normal/High
*Enterobacteriaceae*	≤10^6^ CFU/g	Normal/High
*Citrobacter* spp.	≤10^6^ CFU/g	Normal/High
*Enterobacter* spp.	≤10^6^ CFU/g	Normal/High
*Klebsiella* spp.	≤10^6^ CFU/g	Normal/High
*Proteus* spp.	≤10^6^ CFU/g	Normal/High
*Serratia* spp.	≤10^6^ CFU/g	Normal/High
*Pseudomonas* spp.	≤10^5^ CFU/g	Normal/High
Alpha-haemolytic streptococci	≤10^5^ CFU/g	Normal/High
Beta-haemolytic streptococci	≤10^5^ CFU/g	Normal/High
Acetate	>41.4 μmol/g	Low/Normal
Propionate	>10.2 μmol/g	Low/Normal
Butyrate	>7 μmol/g	Low/Normal
Fecal pH	5.5–6.5	Normal/Abnormal
Beta-defensin	8–60 ng/g	Low/Normal/High
Calprotectin	<50 μg/g	Normal/High
EPX	<1358 ng/g	Normal/High
Secretory IgA	510–2040 μg/g	Low/Normal/High

CFU /g—colony-forming units per gram of feces; SCFA—short-chain fatty acid; EPX—eosinophil protein X; IgA—immunoglobulin A; ≥—greater than or equal to; ≤—less than or equal to.

**Table 2:** Marker-level prevalence of microbiota, functional, and mucosal immune abnormalities.

Marker	Normal, n (%)	Low, n (%)	High, n (%)
1. Core bacterial markers			
*Enterococcus* spp.	34 (34.7)	44 (44.9)	20 (20.4)
*Escherichia coli*	27 (27.6)	28 (28.6)	43 (43.9)
*Bacteroides* spp.	92 (93.9)	6 (6.1)	0
*Enterobacteriaceae*	96 (98.0)	0	2 (2.0)
2. Protective bacterial markers			
*Bifidobacterium* spp.	72 (73.5)	26 (26.5)	0
*Lactobacillus* spp.	56 (57.1)	42 (42.9)	0
3. Fungal markers			
*Candida* spp.	70 (71.4)	0	28 (28.6)
*Candida albicans*	75 (76.5)	0	23 (23.5)
4. Functional markers			
Fecal pH	65 (66.3)	2 (2.0)	31 (31.6)
Acetate	77 (78.6)	21 (21.4)	0
Propionate	81 (82.7)	17 (17.3)	0
Butyrate	88 (89.8)	10 (10.2)	0
5. Mucosal immune and inflammatory markers			
Beta-defensin	53 (54.1)	1 (1.0)	44 (44.9)
Calprotectin	75 (76.5)	0	23 (23.5)
EPX	98 (100.0)	0	0
Secretory IgA	30 (30.6)	48 (49.0)	20 (20.4)

Values are presented as *n* (%). Marker classifications were based on the laboratory reference intervals used for stool microbiota and fecal biomarker analysis. “Low” indicates values below the reference range, whereas “High” indicates values above the reference range. For fungal markers, high values were interpreted as fungal overgrowth according to laboratory thresholds. EPX—eosinophil protein X; IgA—immunoglobulin A.

**Table 3:** Prevalence of dysbiosis domains according to sex.

Dysbiosis Domain	Women, n (%)	Men, n (%)	*p* Value
Core bacterial imbalance	51 (96.2)	36 (80.0)	0.021
Protective bacterial depletion	26 (49.1)	29 (64.4)	0.155
Opportunistic bacterial overgrowth	25 (47.2)	24 (53.3)	0.685
Fungal overgrowth	22 (41.5)	21 (46.7)	0.685
Functional and mucosal immune abnormalities	51 (96.2)	41 (91.1)	0.399

**Table 4:** Prevalence of dysbiosis domains according to place of residence.

Dysbiosis Domain	Urban, n (%)	Rural, n (%)	*p* Value
Core bacterial imbalance	49 (92.5)	38 (84.4)	0.473
Protective bacterial depletion	31 (58.5)	24 (53.3)	0.948
Opportunistic bacterial overgrowth	28 (52.8)	21 (46.7)	0.604
Fungal overgrowth	25 (47.2)	18 (40.0)	0.433
Functional and mucosal immune abnormalities	49 (92.5)	43 (95.6)	0.664

## Data Availability

The datasets presented in this article are not readily available because they are part of an ongoing study. Requests to access the datasets should be directed to Nicoleta Negrut.
